# Recombinant Antibody Fragments for Neurological Disorders: An Update

**DOI:** 10.2174/1570159X21666230830142554

**Published:** 2023-08-31

**Authors:** Karen Manoutcharian, Goar Gevorkian

**Affiliations:** 1 Instituto de Investigaciones Biomedicas, Universidad Nacional Autonoma de Mexico (UNAM), CDMX, Mexico

**Keywords:** Nanobody, intrabody, antibody fragment, Alzheimer’s disease, Parkinson’s disease, Huntington’s disease, immunotherapy

## Abstract

Recombinant antibody fragments are promising alternatives to full-length immunoglobulins, creating big opportunities for the pharmaceutical industry. Nowadays, antibody fragments such as antigen-binding fragments (Fab), single-chain fragment variable (scFv), single-domain antibodies (sdAbs), and bispecific antibodies (bsAbs) are being evaluated as diagnostics or therapeutics in pre-clinical models and in clinical trials. Immunotherapy approaches, including passive transfer of protective antibodies, have shown therapeutic efficacy in several animal models of Alzheimer’s disease (AD), Parkinson’s disease (PD), frontotemporal dementia (FTD), Huntington’s disease (HD), transmissible spongiform encephalopathies (TSEs) and multiple sclerosis (MS). There are various antibodies approved by the Food and Drug Administration (FDA) for treating multiple sclerosis and two amyloid beta-specific humanized antibodies, Aducanumab and Lecanemab, for AD. Our previous review summarized data on recombinant antibodies evaluated in pre-clinical models for immunotherapy of neurodegenerative diseases. Here, we explore recent studies in this fascinating research field, give an update on new preventive and therapeutic applications of recombinant antibody fragments for neurological disorders and discuss the potential of antibody fragments for developing novel approaches for crossing the blood-brain barrier (BBB) and targeting cells and molecules of interest in the brain.

## 1 INTRODUCTION

Recombinant antibody fragments are promising alternatives to full-length immunoglobulins, creating huge opportunities for the pharmaceutical industry. Higher solubility, stability, specificity and affinity, reduced immunogenicity, the ability to refold after heat denaturation, the capacity to target hidden epitopes and cross the blood-brain barrier (BBB), and inexpensive large-scale production are the advantages of antibody fragments compared to conventional monoclonal antibodies [1-14]. In addition, antibody fragments do not induce microglia FcγR-mediated pro-inflammatory response and tissue damage in the CNS because they lack the Fc portion of the immunoglobulin molecule [15, 16].

Nowadays, there is a handful of recombinant antibody fragments approved by the FDA for therapeutic use in individuals with diabetic retinopathy, age-related macular degeneration, Chron's disease, moderate to severely active rheumatoid arthritis, acquired thrombotic thrombocytopenic purpura and thrombosis (The Antibody Society. Therapeutic monoclonal antibodies approved or in review in the EU or US. www.antibodysociety.org/resources/approved-antibodies). Also, many antibody fragments such as antigen-binding fragments (Fab), single-chain fragment variable (scFv) consisting of the antigen-binding domains of Ig heavy (VH) and light (VL) chain regions connected by a flexible peptide linker, single-domain antibody fragments (sdAbs) like camelid heavy-chain variable domains (VHHs) and shark variable new antigen receptor (VNARs), and bispecific antibodies (bsAbs) are being evaluated as diagnostics or therapeutics in pre-clinical models and clinical trials of cancer as well as infectious, neurological, and autoimmune diseases (11, 12, 14, www.clinicaltrials.gov).

Immunotherapy approaches, including passive transfer of protective antibodies and their fragments, have shown therapeutic efficacy in several animal models of Alzheimer’s disease (AD), Parkinson’s disease (PD), frontotemporal dementia (FTD), Huntington’s disease (HD), transmissible spongiform encephalopathies (TSEs) and multiple sclerosis (MS) [17-26]. It has been demonstrated that these molecules may target toxic extra- and intracellular misfolded proteins involved in the pathogenesis of AD, PD, FTD, HD, or TSEs and toxic immune cells participating in the pathogenesis of MS [23, 25, 27-31].

In our previous review, we summarized data on recombinant antibody fragments evaluated until 2016 in pre-clinical models and clinical studies for immunotherapy of neurodegenerative diseases [5]. Here, we explore recent studies in this fascinating research field and give an update on new preventive and therapeutic applications of recombinant antibody fragments for neurological disorders. In addition, we discuss the potential of antibody fragments for developing novel approaches for crossing the blood-brain barrier (BBB) and targeting cells and molecules of interest in the brain.

## 2 ALZHEIMER’S DISEASE (AD)

AD, the most common cause of dementia, is a multifactorial disease and is considered to be a result of several pathological events, both in the periphery and in the brain, including chronic local or systemic inflammation, cerebrovascular dysfunction, oxidative stress, mitochondrial dysfunction, amyloidosis, synaptic and neuronal network dysfunction, metabolic syndrome as well as many disrupted cell signaling pathways [revised extensively elsewhere, 32-35]. The accumulation of extracellular and intracellular amyloid-beta (Aβ) peptide aggregates and neurofibrillary tangles, consisting of the hyperphosphorylated microtubule-associated protein tau, in the human brain has been hypothesized to play a central role in the neuropathology of AD, and efforts have been made to generate antibody-based therapeutics for targeting Aβ and tau aggregates [5, 24, 26, 36, 37]. Here, we discuss recent studies reporting the use of different formats of antibody fragments in *in vivo* pre-clinical models and clinical studies for immunotherapy of AD (Table **1**).

### 2.1 New Studies on Further Characterization and Application of Already Reported Recombinant Antibody Fragments

ScFv-h3D6 is a recombinant antibody fragment derived from a fully humanized and extensively evaluated in clinical trials monoclonal anti-Aβ antibody bapineuzumab. We described this fragment’s development and protective properties in our previous review [5]. Subsequently, the authors modified the scFv-h3D6 by introducing mutations in the VH domain and demonstrated that the improved variants are of high therapeutic interest [38]. Likewise, the authors observed that the scFv-h3D6 inhibits human astrocyte's uptake of toxic Aβ oligomers *in vitro* [39]. Importantly, it has been shown previously that astrocytes accumulate and spread toxic Aβ species because of endosomal/lysosomal fault and inefficient digesting caused by Aβ, leading to microvesicle-induced apoptosis of neurons [40]. Interference in Aβ binding and uptake by astrocytes using scFv-h3D6 antibody may be one of the mechanisms explaining the protective properties of this fragment widely used previously [39]. To improve the production of scFv-h3D6 fragment, the authors used the yeast Pichia pastoris. They demonstrated that scFv-h3D6 obtained in this system could reduce amyloid burden in the 3xTg-AD mouse brain. It does not contain LPS contamination or conformational changes observed in recombinant proteins obtained in *E. coli* [41]. These findings are important for the pharmaceutical industry for large-scale production of scFv fragments that increasingly attract more attention.

Also, the authors further investigated the protective mechanism of scFv-h3D6. They found that it crosses the BBB after a single intraperitoneal (i.p.) injection in 3xTg-AD mice and decreases Aβ levels in the brain [42, 43]. scFv-h3D6 and Aβ were detected in neurons and microglia, suggesting that scFv-h3D6 may cross the cell membrane and that microglia can phagocyte scFv-Aβ complex [42, 43]. Surprisingly, the authors also found endothelial cells with intracellular scFv-h3D6 and Aβ, indicating the involvement of the endothelial cells in the process [42]. Importantly, the authors observed the reduction of intracellular Aβ burden and prevention of neuronal loss [43]. Inflammatory markers in the brain and kidney or liver side effects were not documented in treated animals, and improvement in spatial memory was observed [43]. Then, an *in vivo* longitudinal study using scFv-h3D6 was performed: 5-month-old 3xTg-AD mice received monthly i.p. injections for seven months, and four magnetic resonance imaging and spectroscopy (MRI/MRS) studies were performed at 5, 7, 9, and 12 months of age [44]. Authors documented the reduction in Aβ and proinflammatory cytokines levels in scFv-treated mice compared with control 3xTg-AD mice, and no side effects were observed after multiple doses [44]. When administered to 22-month-old female 3xTg-AD mice by i.p. injection every 3 days for 15 days, scFv-h3D6 antibody ameliorated Aβ burden in the hippocampus, suggesting the therapeutic potential of this fragment in old animals with abundant amyloid accumulation [45]. As in previous studies, scFv-h3D6 co-localized with Aβ inside the CA3 neurons, indicating that it may also reduce intraneuronal Aβ [45]. These findings suggest that the immunotherapy protocols using Aβ-specific scFvs represent a promising therapeutic tool for Aβ elimination without side effects after peripheral injection.

In our previous review, we summarized reports from Dr. Sierks’s group on selecting and characterizing a number of scFvs specifically binding to Aβ, tau, or TDP-43 [5]. Recently, some of these fragments were used to develop blood-based biomarkers for the early diagnosis of AD [46, 47]. The authors analyzed longitudinal human plasma samples from individuals with AD and mild cognitive impairment (MCI) and controls and demonstrated the capacity of scFvs to differentiate AD cases from controls, suggesting that antibody fragments may be used as blood-based biomarkers for AD.

Likewise, two previously reported single-domain camelid VHH antibody fragments, designated PrioAD12 and PrioAD13 and specifically binding to oligomeric Aβ1-40 and Aβ1-42, respectively, were shown to detect Aβ oligomers in the retina and blood of APP/PS1 mice before amyloid accumulation in the brain and before the cognitive decline, pointing to the potential of such nanobodies as early diagnostic tools for AD [48].

### 2.2 Novel Recombinant Antibody Fragments

Two novel camelid single-domain antibodies (VHHs), designated R3VQ and A2 and specifically binding to Aβ and tau, respectively, were shown to cross the BBB, diffuse in the brain after intravenous administration, and bind to extracellular plaques and intracellular tangles [49]. Subsequently, R3VQ VHH was used to generate imaging probes for magnetic resonance imaging (MRI) that were shown to cross the BBB and label Aβ deposits after intravenous injection [50]. These new fragments are very promising for developing diagnostics and therapeutics for AD.

Sebollela and collaborators identified a human scFv, NUsc1, which distinguished Aβ oligomers from monomeric and fibrillar Aβ and reduced oligomeric Aβ-induced oxidative stress and tau hyperphosphorylation in cultured neurons [51]. NUsc1 scFv is also bound to Aβ in brain extracts from APP-transgenic mice and individuals with AD [51]. In addition, NUsc1 scFv recognized Aβ oligomers in fixed AD brain samples [51]. It is known that Aβ oligomers are the most toxic and pathogenic aggregates found in the brain at early stages before the build-up of amyloid plaques, and molecules targeting oligomeric Aβ are of great importance for AD therapy [52]. Subsequently, the authors cloned NUsc1 into the adeno-associated viral vector (AAV) and demonstrated that the treatment with AAV-NUsc1 prevents oligomeric Aβ-induced memory impairment and reverses memory deficits in old APPswe/PS1ΔE9 mice [53]. These results indicate that viral vector-mediated delivery of Aβ-specific antibody fragments is a viable therapeutic approach in Alzheimer's disease. Importantly, nowadays, many AAV vectors are approved by the FDA and are widely used for gene therapy in humans.

Hu and collaborators isolated a novel Aβ-specific scFv17 fragment after screening an immune mouse scFv library and demonstrated its protective properties in APP/PS1 transgenic mice after intracranial injection [54].

Also, efforts have been made to produce and characterize recombinant antibody fragments targeting pathological forms and toxic aggregates of tau. An scFv antibody fragment, designated scFv-MC1 and based on the variable heavy (VH) and variable light (VL) chains of the well-characterized anti-tau monoclonal antibody MC1, was constructed, cloned into the AAV, and shown to reduce tau pathology after intracranial injection in adult JNPL3 transgenic mice, expressing human P301L-mutant tau [55]. Subsequently, the authors demonstrated that a single intramuscular injection of AAV1-scFvMC1 significantly reduces insoluble and soluble tau aggregates in the brain of two different tau transgenic mice, JNPL3 and P301S [56]. The authors detected scFvMC1 in the microglia, and, importantly, no inflammatory molecules were detected in the brain, suggesting the therapeutic potential of anti-tau scFv for AD and other tauopathies without side effects [56]. AT8-scFv fragment, based on the VL and VH regions of another well-characterized anti-phospho-Tau (Ser202, Thr205) monoclonal antibody AT8, was cloned into the AAV and shown to cross the BBB, infect neurons and express AT8-scFv for at least 22 weeks [57]. Two other tau-specific single-domain antibodies, VHH-E4-1, and its improved variant Z70, binding to the microtubule-binding domain (MTBD) of tau were shown to prevent tau fibrils assembly *in vitro* and inhibit tau seeding in the transgenic mouse model THY-tau 30 [58].

It has been reported that alternative splicing generates two major isoforms of tau containing either 3 or 4 repeats (R) segments, and both forms are detected in the brain of AD and other tauopathies patients; however, few studies were dedicated to the development of therapeutic strategies for targeting these toxic aggregates [59]. A scFv antibody designated 5F10 and selectively binding to the 3Rtau has been developed and fused to the LDL receptor-binding domain of apolipoprotein B (apoB) for enhancing the BBB crossing [60]. The authors demonstrated that the 5F10 antibody reduces the 3RTau accumulation and ameliorates behavioral deficits in a transgenic mouse model of tauopathy; notably, these mice accumulate intraneuronal 3RTau in the hippocampus and neocortex mimicking the accumulation in the AD brain [60].

To decrease the amyloidogenic processing of APP, various β-site amyloid precursor protein cleaving enzyme 1 (BACE-1) inhibitors have been explored as potential disease-modifying drugs for AD [61]. Likewise, a single chain antibody VHH-B9 has been isolated from an immune anti-human BACE-1 phage display VHH library and shown to effectively inhibit neuronal BACE-1 in primary neuronal cultures and reduce astrogliosis and microgliosis, Aβ burden, and cognitive deficits in an APP/Tg mouse model of AD after systemic AAV-mediated delivery [62].

An interesting approach has been developed recently by Dr. Sierks’s group. Since previous studies have shown that selectively inhibiting BACE-1 processing of APP and increasing non-amyloidogenic processing of APP by promoting α-secretase cleavage might have therapeutic importance for AD [63, 64], Sierks and collaborators generated a bispecific recombinant antibody fragment, diabody, combining a scFV, named iBsec1 and binding to BACE-1, and another scFv, named Asec1a that has been shown to promote non-amyloidogenic processing of APP [65]. The obtained bispecific diabody, after fusion with the low-density lipoprotein receptor-binding domain of the apolipoprotein B for enhancing the transfer across the BBB, was capable of reducing amyloid burden in APP/PS1 transgenic mice [65]. In addition, the authors documented increased survival of mice treated with the diabody while mice treated with iBsec1 showed no change in total mortality, suggesting that the simultaneous inhibition of BACE-1 and stimulation of the α-secretase processing of APP may be a more advantageous therapeutic approach for AD [65].

Recently, addressing the multifactorial origin of AD, Zhao and collaborators developed a multivalent nanobody conjugate PNBIL by linking an Aβ-specific nanobody covalently with a rigid, ROS scavenging scaffold poly(deca-4,6-diynedioic acid) [66]. The recombinant nanobody had an Aβ33-42 peptide sequence grafted into the third complementary-determining region (CDR3) and a fragment of human interleukin-1β, thus allowing the binding to the amyloid aggregates, inhibition of Aβ aggregation and effective Aβ clearance by microglia [66]. In addition, the ROS scavenging scaffold confers a long-term inhibition of oxidative stress and inflammation [66]. The protective potential of this multi-target construct has been confirmed in two APP/Tg mouse models of AD [66].

## 3 PARKINSON’S DISEASE (PD)

PD is the second most common neurodegenerative disease, and, as in the case of AD, multiple genetic and environmental factors, including oxidative stress, mitochondrial dysfunction, inflammation, gut dysbiosis, glymphatic system impairment, and the accumulation of pathological aggregates of α-synuclein (α-syn) in the Lewy bodies and Lewy neurites with the subsequent progressive loss of dopaminergic neurons in the brain, were described [revised extensively elsewhere, 34, 67-70]. Immunotherapeutic approaches targeting toxic α-syn aggregates are currently being evaluated in pre-clinical models [67, 71, 72]. All the above-discussed properties of recombinant antibody fragments and the possibility of intracellular expression make them suitable candidates for future therapies for PD.

After publishing our previous review [5], new anti-α-syn antibody fragments were generated and evaluated *in vitro* and *in vivo*. Two scFvs, scFv-pF and scFv-pC, were constructed using the VH and VL sequences of a previously described conformation-specific anti-α-syn monoclonal antibody (Syn-F2) [73]. The authors showed that both scFvs specifically bind to α-syn fibrils and oligomers but not to monomers and detected intracellular aggregates in the brain from individuals with Lewy body pathology [73]. Furthermore, scFv-pF and scFv-pC were capable of inhibiting the seeding of α-syn aggregation and reducing α-syn toxicity in a SH-SY5Y cell model of PD [73]. In addition, the authors developed an improved method for purifying antibody fragments from inclusion bodies and demonstrated its feasibility for the scFv-pF, shown to bind to the α-syn C-terminal region [74].

Fassler and collaborators isolated a novel scFv antibody designated sMB08 after the screening of an antibody fragment library against human α-syn and demonstrated that the sMB08 antibody fragment binds with a high affinity to both human and mouse α-syn oligomers and pre-formed fibrils (PFF), detects α-syn in the substantia nigra and cortex of patients with PD, inhibits α-syn aggregation *in vitro*, inhibits the toxicity of α-syn oligomers and PFF in SH-SY5Y cells, enters neurons and co-localize with α-syn aggregates, attenuates uptake of α-syn oligomers and PFF in human PBMCs and mouse microglia and down-regulates in a dose-dependent manner pro-inflammatory cytokines (TNF-α, IL-6) expression by microglia after exposure to PFF [75]. After intranasal administration, the sMB08 antibody fragment attenuated neuroinflammation in mice that received an intracranial injection of α-syn PFF [75]. Likewise, sMB08 attenuated motor dysfunction after intranasal administration in the rotenone rat model of PD [75].

A single domain antibody Nbα-syn01 was selected after screening an immune camelid VHH phage display library against monomeric α-syn [76]. The authors demonstrated that Nbα-syn01 binds to the N-terminal region of α-syn peptide, known to participate in α-syn aggregation *in vitro* and *in vivo* [76]. Also, Nbα-syn01 had a higher affinity towards α-syn fibrils than a monomeric form, detected Lewy bodies in brain samples from individuals with PD, and inhibited α-syn aggregation and toxicity in SH-SY5Y cells [76].

Mutations in the gene coding for leucine-rich repeat kinase 2 (LRRK2) have been linked with the inherited forms of PD, and the protein is considered an attractive target for immunotherapy of the disease [77-79]. This enzyme has two catalytic activities: GTPase activity mediated by the Roc domain and Ser/Thr protein kinase activity; the latter is considered particularly interesting for the drug development for PD [80]. Although ATP-competitive inhibitors are currently approved by FDA, long-term inhibition of LRRK2 with these molecules was associated with toxic side effects, including kidney and lung abnormalities [80, 81]. Therefore, designing molecules that bind outside the ATP-binding pocket represents an interesting and promising approach. With this hypothesis in mind, Singh and collaborators isolated nanobodies binding to a different region of the LRRK2 and capable of inhibiting the kinase activity in human embryonic kidney 293 (HEK293) cells overexpressing LRRK2 [81].

## 4 HUNTINGTON’S DISEASE (HD)

HD is an autosomal dominant inherited progressive neurodegenerative disorder caused by a trinucleotide repeat expansion in the huntingtin gene resulting in polyglutamine repeats-bearing mutant huntingtin (mhtt) accumulation in the neuronal nuclei and processes and subsequent neuronal loss [82, 83]. Recombinant intracellular antibody-mediated modulation of toxic mhtt aggregates represents an alternative therapeutic approach for HD [5, 30, 84, 85]. In our previous review, we summarized studies reporting various htt-targeting antibody fragments tested *in vitro* and *in vivo* [5]. Subsequently, new fragments were developed and explored. Amaro and Henderson selected a single-chain intrabody, INT41, that bound to the proline-rich region (PRR) of huntingtin and demonstrated that it could inhibit aggregation of transiently overexpressed mhtt in HEK293T cells [86]. After cloning into the AAV6 vector, the rAAV6-INT41 was administered bilaterally into the striatum of 5-week-old R6/2 mice, a transgenic mouse model widely used to study HD [86]. The authors observed that INT41 ameliorates mhtt-induced cognitive impairment and reduces aggregate formation in the striatum of treated animals [86].

## 5 NOVEL PLATFORMS

To enhance immunotherapy efficacy, the development of new platforms for therapeutic antibody application has been the primary goal of scientists and the pharmaceutical industry in recent years.

Perche and collaborators demonstrated that intracranial injection of three different Aβ-specific scFV mRNA polyplexes (cationic polymer-based complexes) significantly decreases Aβ accumulation in an acute amyloidosis model but not in a transgenic model of AD [87]. The authors observed that the mRNA-loaded polyplex nanomicelle platform allows efficient transfection of primary neurons and leads to detectable scFv mRNA expression in the brain during 48h. These results are interesting for further development of mRNA-based immunotherapeutic protocols for neurological disorders.

Another interesting approach has been proposed by Xie and collaborators [88]. In the Fab region of the above-described 3D6 monoclonal anti-Aβ antibody, positively charged primary amino groups were coupled with citraconic anhydride (Cit) through an amidation reaction, resulting in a negatively charged citraconylated-Fab (Cit-Fab) [88]. Then this charge-converted anionic Fab antibody fragment was coupled with a cationic poly(ethylene glycol)-poly(l-lysine) (PEG-PLL) block copolymer giving polymeric nanomicelles (PMs) [88]. The authors showed that 41-fold enhanced brain accumulation was achieved after peripheral injection of 3D6-Fab PMs compared with free 3D6-Fab [88]. In addition, 3D6-Fab PMs were capable of inhibiting Aβ1-42 aggregation in APP/Tg mice [88]. These findings are very promising because the use of nanomicelles may allow reducing substantially the amount of recombinant antibody dose and the frequency of application in patients.

Because the BBB is an obstacle to antibody passage into the brain, efforts were made to overcome it [89]. An interesting approach has been developed where anti-transferrin receptor (TfR) antibody fragments were used as a shuttle for the transport of molecules of interest across the BBB by receptor-mediated transcytosis [90-93]. In an elegant study, Hultqvist and collaborators fused two scFvs, binding to TfR, to the light chains of protofibril-selective anti-Aβ mAb158 and demonstrated that the fusion protein designated mAb158-scFv8D3 enters the brain after peripheral administration in APP-transgenic mice [90]. Fusion protein concentrations were 80-fold higher than those of unmodified mAb158. Notably, after three days, fusion protein concentrations in APP-transgenic mouse brains were 9-fold higher than in non-transgenic mice, suggesting that the fused antibody fragment bound specifically to soluble amyloid protofibrils in the brain [90]. Next, the radiolabeled ^124^I-RmAb158-scFv8D3 antibody fragment has been applied for PET imaging and shown to be a more sensitive reagent for detecting soluble Aβ protofibrils and monitoring the progression of the amyloid pathology compared to the small-molecule ligand 18F-florbetapir that binds to fibrillar amyloid plaques [91]. Subsequently, the authors designed another recombinant antibody fragment, named tribody and composed of two scFvs of the above-mentioned protofibril-selective mAb158 and Fab of anti-TfR monoclonal antibody 8D3 [94]. They demonstrated that such fragments, after labeling with iodine-124, may be used for PET imaging of amyloid aggregates [94]. Also, the authors generated a di-scFv 3D6-8D3 fragment and used it as a PET radioligand for visualization of Aβ in the brain of APP-transgenic mice [92]. Recently, the authors constructed multivalent antibodies using various numbers of heavy and light chains variable fragments of mAb158 and scFv8D3 and demonstrated that bispecific-multivalent recombinant antibodies were capable of crossing the BBB and reducing the amyloid load in 10-11-month-old Tg-ArcSwe mice after intravenous injection [95]. In 2023, the FDA granted accelerated authorization to the humanized version of the mAb158, lecanemab, for use in individuals with mild to moderate AD. Employing the above-mentioned strategies may enhance efficacy and reduce the cost of treatment with lecanemab.

Likewise, a single domain shark antibody VNAR fragment (TXB2), binding to both murine and human TfR1, was fused with the known anti-amyloid-β (Aβ) antibody, bapineuzumab (Bapi) [96]. After intravenous injection of the Bapi-anti-TfR VNAR fusion protein in APP-transgenic mice, Bapi was detected in the brain [96]. In addition, the authors fused another TfR1-specific VNAR, TXB4, to a tropomyosin receptor kinase B (TrkB) receptor agonist antibody and documented reduced neuronal loss in a mouse model of PD after intravenous injection [97]. TXB4-anti-TrkB recombinant antibody has been detected in TrkB-positive cells in the cortex and tyrosine hydroxylase (TH)- positive dopaminergic neurons in the substantia nigra pars compacta (SNc) [97]. These results indicated that anti-TfR1 VNARs may be successfully fused to therapeutic recombinant antibody fragments allowing them to cross BBB [96]. Likewise, an anti-human TfR VHH (Nb188) was selected from an immune anti-human TfR camelid VHH library, fused with neurotensin, and shown to induce hypothermia after intravenous injections in mice [98]. In the same report, the authors fused two anti-human TfR VHHs, Nb62 and Nb88, with anti-BACE-1 antibodies and demonstrated that both fusion proteins could reduce Aβ1-40 levels in the brain after systemic administration [98].

Also, single-domain fragments specifically binding to another receptor on BBB endothelial cells, insulin-like growth factor 1 receptor (IGF1R), were selected from an immune camelid VHH library and shown to bind to brain endothelial cells from different species: humans, rats, and mice [99]. The authors demonstrated that anti-IGF1R VHH fragments carrying conjugated galanin or neurotensin could induce a dose-dependent reduction in hyperalgesia and the core temperature, respectively [99, 100].

Rabbit single-domain VLL antibodies isolated from an immune anti-mouse bEnd. Three endothelial cells were conjugated with liposomes encapsulating a histone deacetylase inhibitor (HDACi) PAN and shown to cross the BBB after intravenous injection in the tail vein of CD1 mice [101].

Superparamagnetic iron oxide nanoparticles (SPIONs) have been explored for numerous biomedical applications, including drug delivery, tissue targeting and repair, iron replacement therapies, and contrast probes in MRI [102]. Liu and collaborators conjugated an Aβ oligomer-specific scFv antibody (W20) with SPIONs to develop an MRI contrast probe and demonstrated that W20-SPIONS could cross the BBB and specifically bind to oligomers in the transgenic mouse model of HD and PD [103]. Subsequently, the authors added the heptapeptide XD4, shown to activate the class A scavenger receptor (SR-A) on the microglia and promote phagocytosis of oligomeric Aβ, and evaluated the protective properties of the conjugate in APPswe/PS1dE9 transgenic mice. W20/XD4-SPIONs nanoparticles significantly ameliorated cognitive deficits and reduced Aβ burden, astrocytosis, microgliosis, and reactive oxygen species levels in the brains of treated mice [104, 105].

All these findings suggest that recombinant antibody fragments, alone or in the context of different nanoparticles, are promising tools for drug delivery into the brain.

## CONCLUSION

Recombinant antibody fragments are promising alternatives to full-length immunoglobulins, creating big opportunities for the pharmaceutical industry. Many fragments in different formats (Fab, scFV, VHH, VLL, VNAR, *etc*.) are currently being evaluated in preclinical models and clinical trials. Recombinant antibody fragments have many advantages over other active and passive immunotherapy approaches. Their high specificity, superior affinity, and improved capacity to cross the BBB make it possible to use them at low concentrations, thus reducing adverse side effects observed frequently in patients after traditional immunotherapy. In addition, the lack of Fc-mediated activation of the peripheral immune cells and the microglia minimizes the risk of undesirable inflammatory response. Likewise, recombinant antibody fragments generally are not glycosylated, allowing their production in time- and cost-effective bacterial and yeast expression systems. All these characteristics make recombinant antibody fragments attractive molecules for the biotech industry for the development of novel diagnostic and therapeutic strategies for neurological disorders. The discovery of new tools for enhanced delivery of antibody fragments into the brain, the design of fusion peptides/proteins for targeting them to the cells/cell compartments of interest, and the application of AAV vector-mediated antibody gene therapy give us hope that in the nearest future, recombinant antibody fragments may become available for the prevention and treatment of several neurological disorders.

## Figures and Tables

**Table 1:** Summary of recombinant antibody fragments used in AD, PD and HD pre-clinical models.

**Recombinant Ab Fragment**	**Target/Disease**	**Key Findings**	**References**
Purified scFv-h3D6	Aβ, Alzheimer’s disease	Affinity maturation for enhanced binding. Inhibition of Aβ uptake by human astrocytes. Use of Pichia pastoris for improved production. Co-localization with Aβ in endothelial cells. Attenuation of amyloid pathology and cognitive impairment in APP-Tg mice.	[38] [39] [41] [42] [43-45]
Purified scFvs: C6T, E1, D11C, ADTau2, ADTau4, ADTau6, AD-TDP3	Aβ, Tau, TDP-43 Alzheimer’s disease	Multiparameter blood biomarker panel for the early diagnosis of AD.	[46, 47]
Purified camelid VHHs: PrioAD12, PrioAD13	Aβ, Alzheimer’s disease	Early detection of Aβ in the retina and blood samples.	[48]
Purified VHH: R3VQ	Aβ, Alzheimer’s disease	Crosses the BBB and binds to amyloid plaques. Suitable for MRI.	[49, 50]
Purified VHH: A2	Tau, Alzheimer’s disease	Crosses the BBB, diffuses in the brain and binds to intraneuronal tangles.	[49]
AAV-expressed scFv NUsc1	Aβ, Alzheimer’s disease	Prevents oligomeric Aβ-induced memory impairment and reverses memory deficits in APP-Tg mice.	[51-53]
scFv17	Aβ, Alzheimer’s disease	Reduces Aβ load in the brain of APP-Tg mice.	[54]
AAV-expressed scFv-MC1	Tau, Alzheimer’s disease and other Tauopathies	Reduces tau pathology in Tg mice.	[55, 56]
AAV-expressed AT8-scFv	Tau, Alzheimer’s disease and other Tauopathies	Crosses the BBB and enters neurons.	[57]
VHH-E4-1 and VHH-Z70	Tau, Alzheimer’s disease and other Tauopathies	Inhibit Tau seeding in Tg mice.	[58]
AAV-expressed scFv-EM-48	Tau, Alzheimer’s disease and other Tauopathies	Reduces Tau accumulation and ameliorates behavioral deficits in Tg mice.	[59, 60]
AAV-expressed VHH-B9	BACE-1, Alzheimer’s disease	Reduces Aβ burden and cognitive deficits in Tg mice.	[62]
Bispecific diabody, iBsec1-Asec1a	BACE-1, α-secretase, Alzheimer’s disease	Reduces Aβ burden and increases the survival of mice.	[65]
Purified scFv-pF and scFv-PC. Purified VHH Nbα-syn01	α-syn, Parkinson’s disease	Inhibit the seeding of α-syn aggregation and reduce α-syn toxicity in SH-SY5Y cells.	[73-75]
Purified scFv sMB08	α-syn, Parkinson’s disease	Enters neurons and co-localizes with α-syn aggregates. Attenuates motor dysfunction in rats.	[75]
Purified VHH Nbα-syn01	α-syn, Parkinson’s disease	Inhibit the seeding of α-syn aggregation and reduce α-syn toxicity in SH-SY5Y cells.	[76]
AAV-expressed scFv INT41	mhtt, Huntington’s disease	Ameliorates mhtt-induced cognitive impairment and reduces aggregate formation in the striatum of treated mice.	[86]

## LIST OF ABBREVIATIONS

AAV: Adeno-associated Viral Vector
AD: Alzheimer’s Disease
Aβ: Amyloid-beta
BBB: Blood-brain Barrier
FDA: Food and Drug Administration
FTD: Frontotemporal Dementia
HD: Huntington’s Disease
HDACi: Histone Deacetylase Inhibitor
IGF1R: Insulin-like Growth Factor 1 Receptor
MCI: Mild Cognitive Impairment
MRI: Magnetic Resonance Imaging
MRI/MRS: Magnetic Resonance Imaging and Spectroscopy
MS: Multiple Sclerosis
MTBD: Microtubule-binding Domain
PD: Parkinson’s Disease
PMs: Polymeric Nanomicelles
PRR: Proline-rich Region
scFv: Single-chain Fragment Variable
SPIONs: Superparamagnetic Iron Oxide Nanoparticles
TSEs: Transmissible Spongiform Encephalopathies
VNARs: Variable New Antigen Receptor
